# Comparison of serum fatty acid content and caloric expenditure after a single bout of moderate-intensity and high-intensity treadmill exercise in young females

**DOI:** 10.1186/1550-2783-11-S1-P24

**Published:** 2014-12-01

**Authors:** Jordan Outlaw, Sara Hayward, Josh Holt, Bailey Burks, Eliza Faillace, Alena Regelski, Matt Stone, Meagon Sauer, Katelyn Villa, Jacy Mullins, Stacie Urbina, Lem Taylor, Cliffa Foster, Colin Wilborn

**Affiliations:** 1 University of Mary Hardin-Baylor, Belton, Texas, USA

## Background

The idea behind high-intensity interval training (HIIT) is that an individual will burn a greater amount of calories and fat in a shorter period of time than moderate-intensity exercise. While beneficial changes have been seen when HIIT is incorporated into training programs, there is still information about HIIT that is unknown. At moderate intensities, triacylglycerol is the predominate fuel source based on percentage while carbohydrates are the predominate fuel during high-intensity exercise. Serum fatty acid (FFA) can be used as marker of fat utilization during and after exercise. The purpose of this investigation was to determine the amount of FFA released during moderate-intensity and high-intensity treadmill running in endurance trained females.

## Methods

Seven female runners (VO2MAX ≥42.0 ml/kg/min) volunteered to participate in this study and completed a baseline VO2MAX treadmill test and a dual-energy x-ray absorptiometry scan (DEXA) to determine fitness level and body composition. Participants then completed either a 45-minute run at 6mph or 10 30-second sprints with 30 seconds of rest between each sprint. After seven days, the participants returned to perform the other running protocol, in a crossover manner. While performing both protocols, participants were connected to the TrueOne ParvoMedics metabolic cart to determine macronutrient utilization (percent) and caloric expenditure. Blood was drawn immediately before (0min), 30-minutes post (30min), and 60-minutes (60min) post-exercise and analyzed for serum free fatty acids. Data were analyzed using a repeated measures ANOVA [2 (group) x 3 (time)] and [2 (group) x 2 (time)], and one-way ANOVA for calorie expenditure. Significance was set at p ≤ 0.05. Consent to publish the results was obtained from all participants.

## Results

Both groups had a significant linear increase in FFA from 0min to 30min (p = 0.028), 30min to 60min (p = 0.05), and 0min to 60min (p = 0.014). There was a significant difference between groups from 0min to 60min (p = 0.005) with the run having a greater amount of FFA in the blood compared to the sprints. There was a significantly greater increase in FFA for the run for 0min to 30min (p = 0.003) but no difference from 30min to 60min (p = 0.507) compared to the sprints. RER was significantly greater for sprinting (p = 0.014) compared to running. The percent carbohydrate usage was significantly greater for sprinting (p = 0.007) and percent fat usage significantly greater for running (p = 0.007) when comparing running and sprinting. There was no significant difference between protocols for caloric expenditure per minute (p = 0.515).

## Conclusion

The results from this investigation indicate that both moderate- and high-intensity running result in significant increases in FFA with moderate-intensity running having a significantly greater increase in FFA from immediately before to 30 minutes post-exercise.

